# Supplementary material for: Unveiling the Multifaceted Dynamics of Breast Cancer: A Copula Regression Approach to Modeling and Predicting Outcomes
Source: PLoS One. 2026 Apr 10;21(4):e0346495. doi: 10.1371/journal.pone.0346495 (PMC13068339; doi:10.1371/journal.pone.0346495)
Supplement: S1 Table — Normal, Gamma, Log-normal, Logistic, and Inverse-Gamma distributions were fitted to the data. The Normal distribution provided the best fit based on AIC and BIC. (PDF) [file pone.0346495.s001.pdf]

S1 Table: Comparison of AIC and BIC values for candidate marginal distributions of Age at Diagnosis

**Distribution**	**AIC**	**BIC**
Normal	15167.40*	15178.51*
Gamma	15261.90	15272.90
Log-normal	15351.19	15362.29
Logistic	15258.55	15269.66
Inverse-gamma	15472.02	15483.12

Note: AIC = Akaike Information Criterion, BIC = Bayesian Information Criterion. Lower values indicate better fit. The Normal distribution showed the lowest AIC and BIC values (indicated by *), confirming it as the most appropriate marginal distribution for age at diagnosis in the METABRIC cohort.
