# Supplementary figures and images for: Unveiling the Multifaceted Dynamics of Breast Cancer: A Copula Regression Approach to Modeling and Predicting Outcomes
Source: PLoS One. 2026 Apr 10;21(4):e0346495. doi: 10.1371/journal.pone.0346495 (PMC13068339; doi:10.1371/journal.pone.0346495)

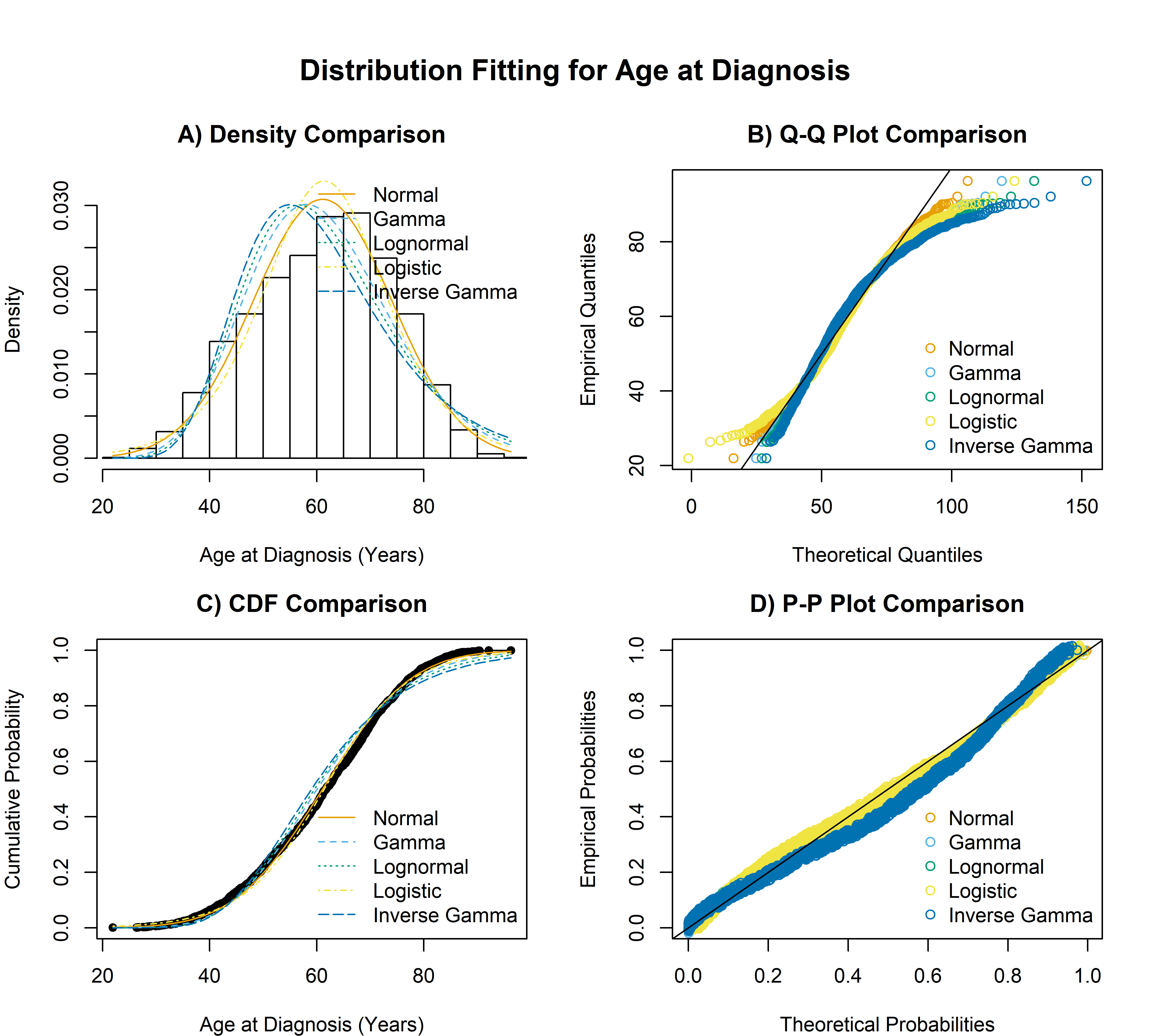

Supplement: S1 Fig — Normal, Gamma, Log-normal, Logistic, and Inverse-Gamma distributions were fitted to the data. Visual inspection confirmed the Normal distribution as the most appropriate choice. (TIFF) [file pone.0346495.s002.tiff]

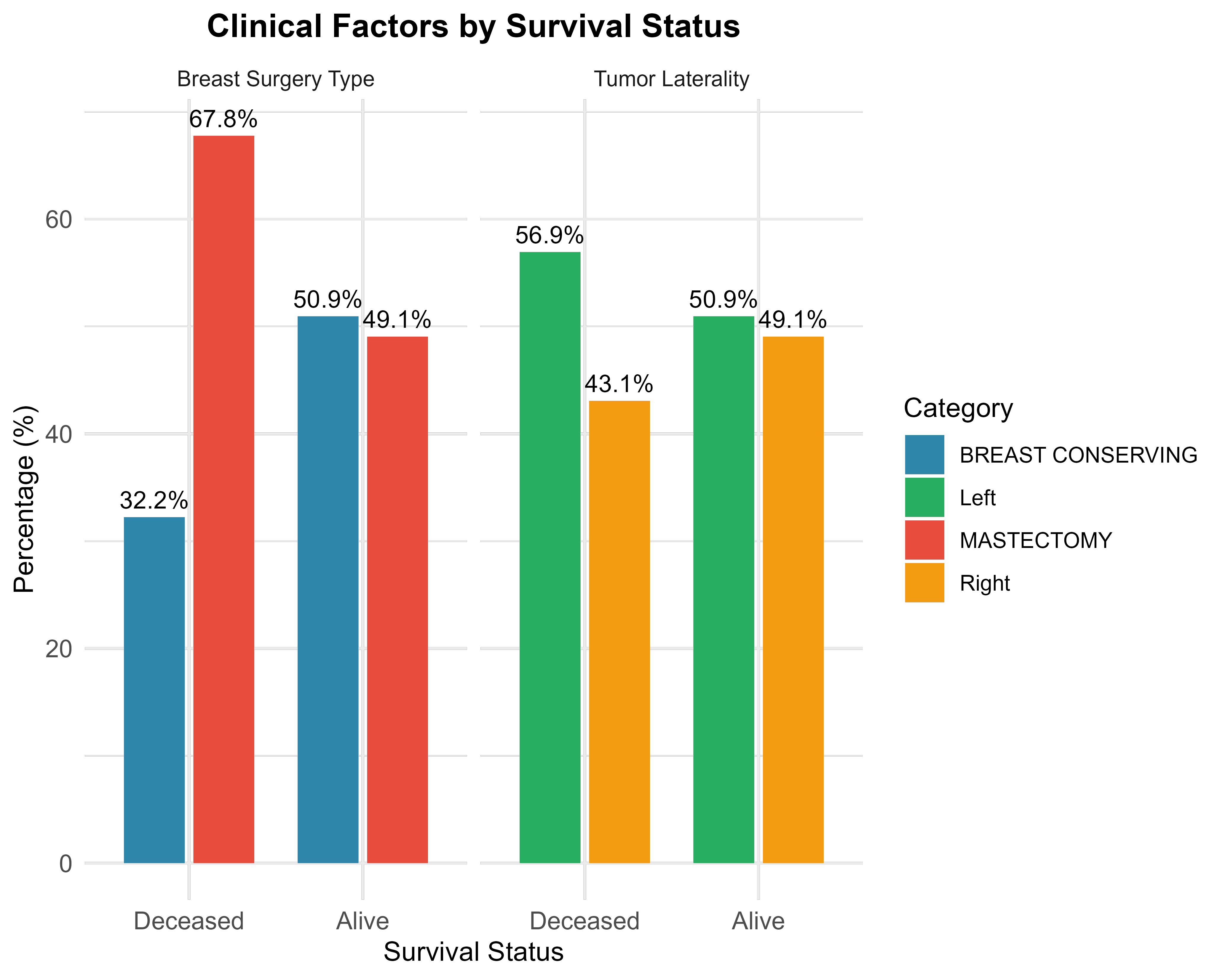

Supplement: S2 Fig — Combined bar plots depict the distribution of breast-conserving surgery versus mastectomy and tumor laterality (left vs. right breast) among surviving and deceased patients. Distinct patterns suggest potential associations between surgical choice, tumor location, and patient survival. (TIFF) [file pone.0346495.s003.tiff]

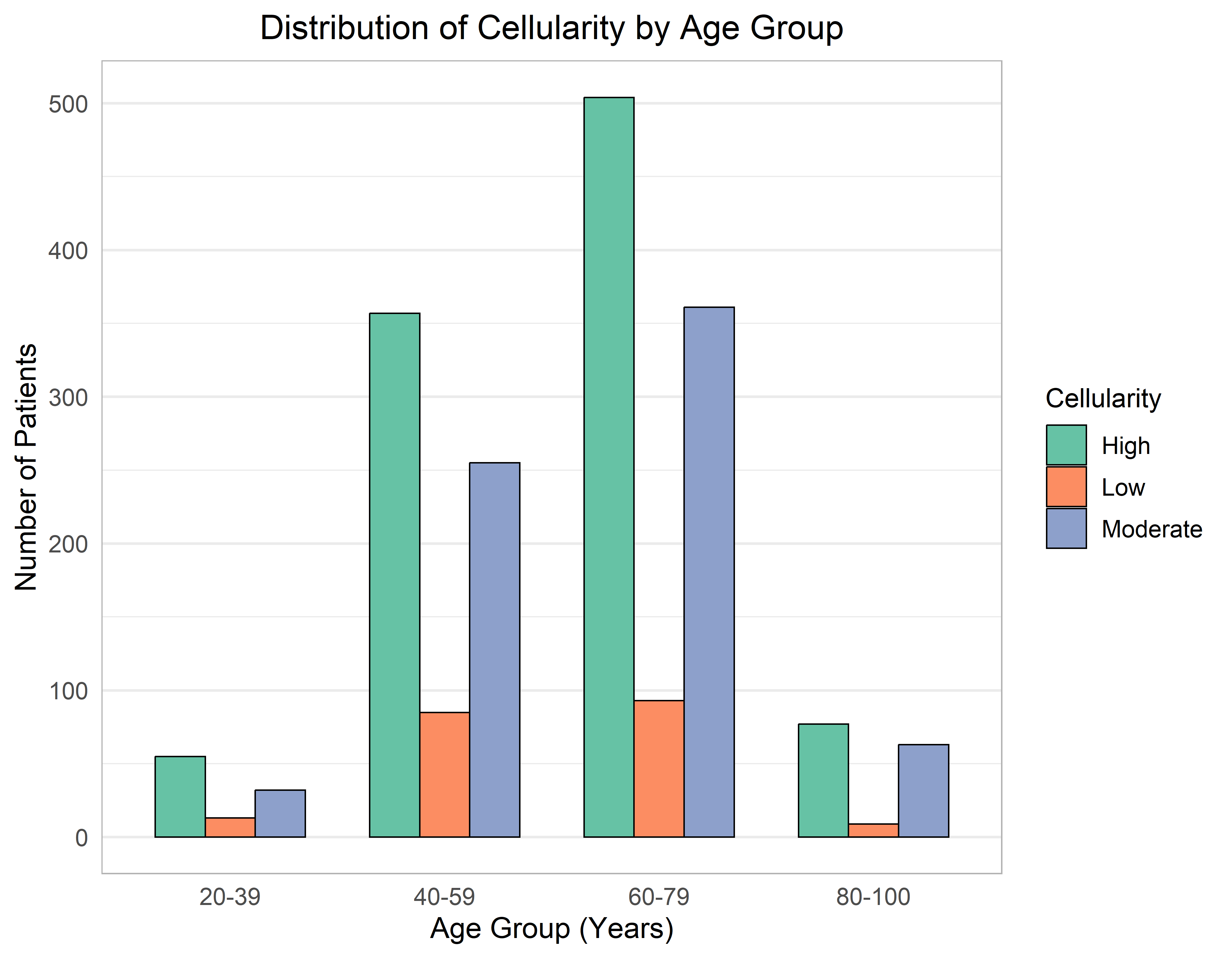

Supplement: S3 Fig — Grouped bar charts show how cellularity (low, moderate, high) varies across age categories. Older patients more frequently exhibited higher cellularity levels, indicative of more aggressive disease profiles. (TIFF) [file pone.0346495.s004.tiff]

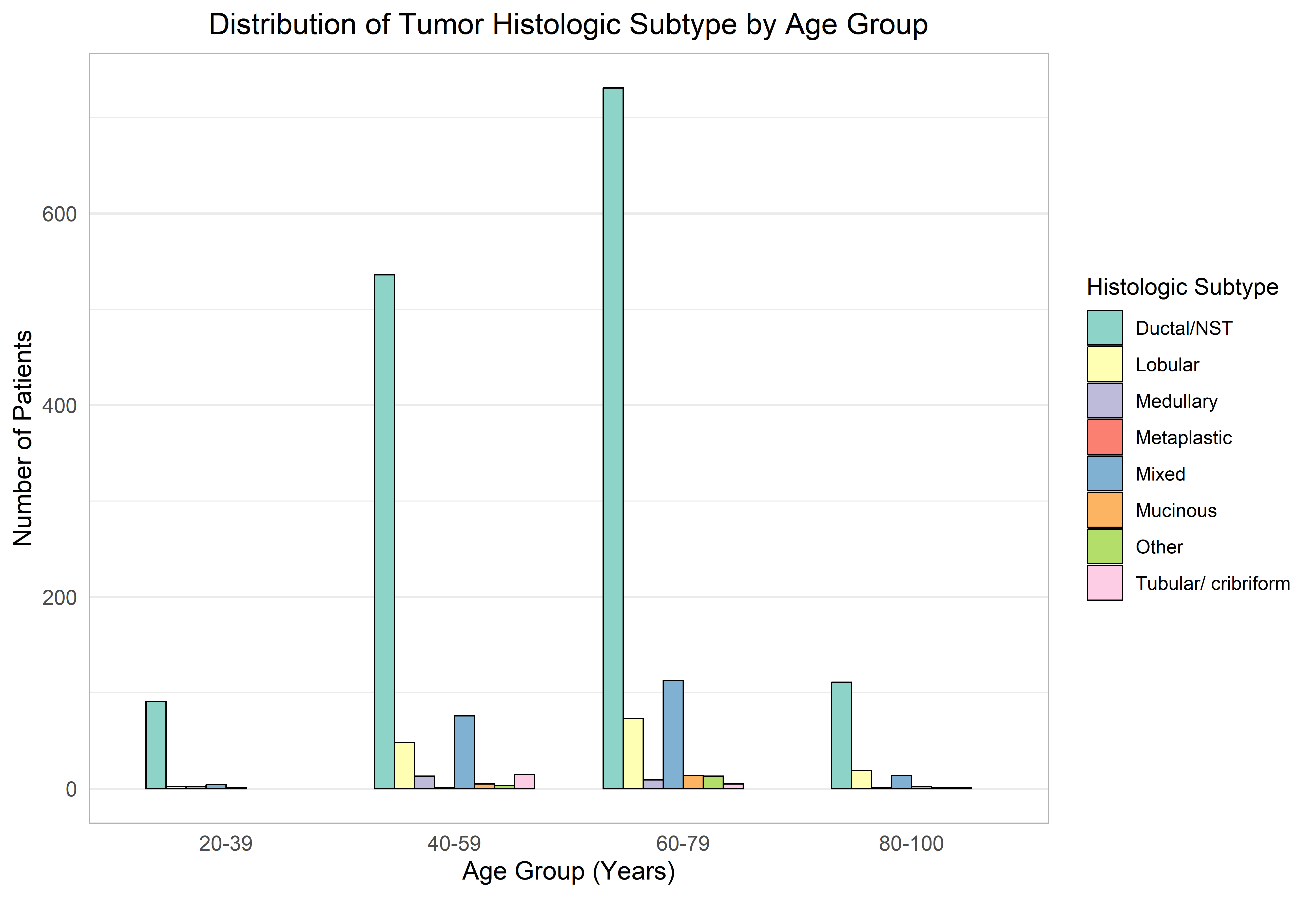

Supplement: S4 Fig — Bar plots demonstrate how integrative molecular cluster frequencies vary across age categories. Differences in cluster prevalence with age suggest underlying biological and genomic heterogeneity within the cohort. (TIFF) [file pone.0346495.s005.tiff]

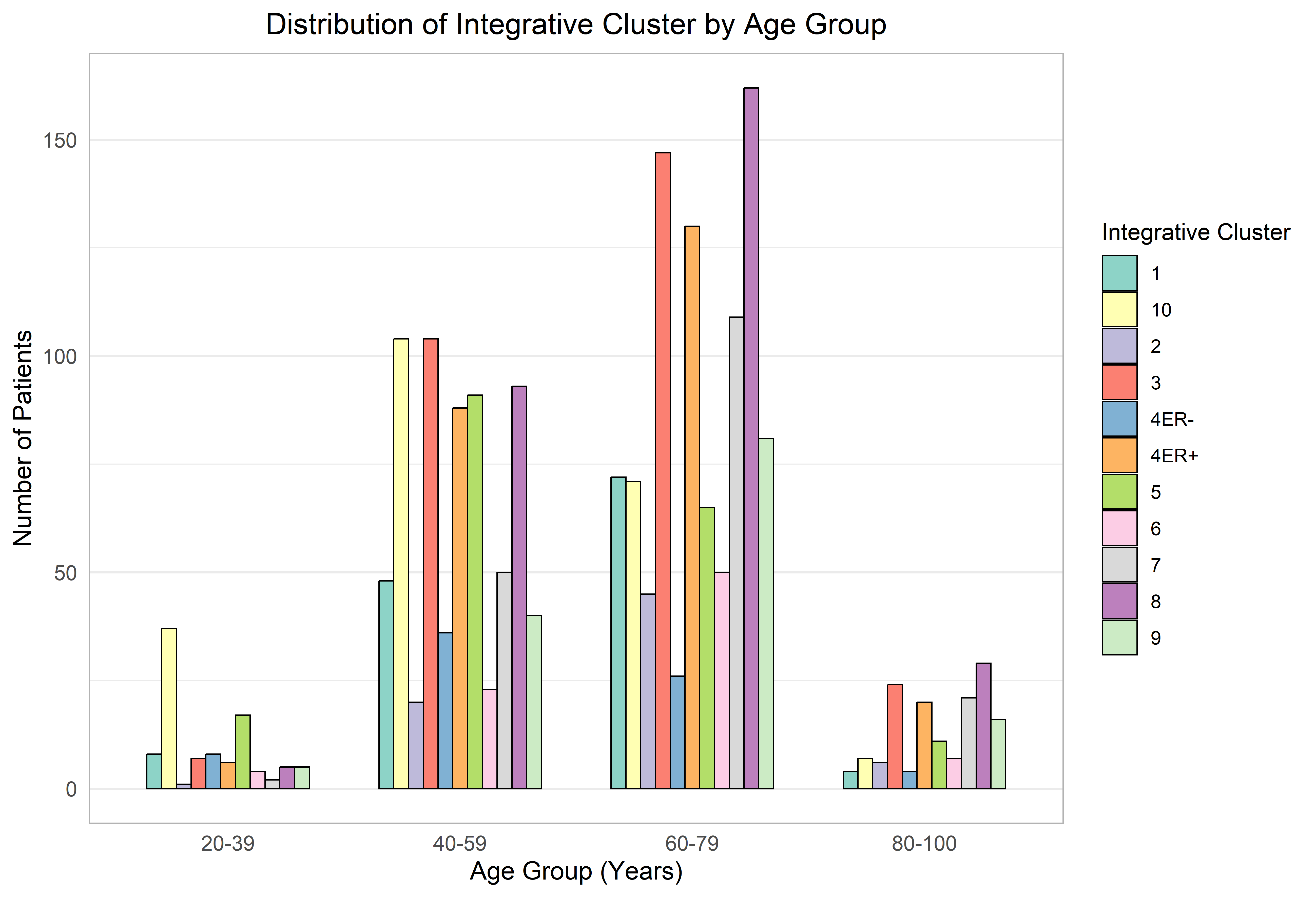

Supplement: S5 Fig — The distribution of ductal/NST, lobular, and mixed histologic subtypes is shown by age category. While ductal/NST carcinoma remains predominant in all age groups. (TIFF) [file pone.0346495.s006.tiff]
