# Supplementary material for: Unveiling the Multifaceted Dynamics of Breast Cancer: A Copula Regression Approach to Modeling and Predicting Outcomes
Source: PLoS One. 2026 Apr 10;21(4):e0346495. doi: 10.1371/journal.pone.0346495 (PMC13068339; doi:10.1371/journal.pone.0346495)
Supplement: S1 Appendix — This appendix provides the full technical derivation and computational details for the mixed binary-continuous copula models described in the main text. (PDF) [file pone.0346495.s007.pdf]

Supplementary Appendix: Comprehensive Technical Details for the Copula Models

S1 Appendix. Comprehensive Technical Details for the Copula Models.This appendix provides the full technical derivation and computational details for the mixed binary-continuous copula regression model described in the main text. The content here is intended for readers interested in implementation details, reproducibility, or methodological extensions. We follow the approach of Klein et al. [2019] and Marra and Radice [2017].

A1. Complete Model Specification

Parameter Vector and Regression Framework The complete parameter vector for our Bernoulli-Gaussian copula model is $\boldsymbol{\vartheta}_i = (\pi_i, \mu_i, \sigma_i, \theta_i)^T$, where:

- π_i: Probability of success (survival) for the binary outcome
- μ_i: Mean of the continuous outcome (age at diagnosis)
- σ_i: Standard deviation of the continuous outcome
- θ_i: Copula dependence parameter

The total number of parameters to estimate is $Q = Q_1 + Q_2 + Q_c = 1 + 2 + 1 = 4$ per observation, before considering covariate effects.

Regression Predictor Specification Each distributional parameter ϑ_k is linked to covariates through a structured additive predictor:

$$\gamma_{ik} = g_k(\vartheta_{ik}) = \beta_0^{\vartheta_k} + \sum_{l=1}^{L_k} M_l^{\vartheta_k}(\boldsymbol{\xi}_i)$$

where $g_k(\cdot)$ is the link function, $\beta_0^{\vartheta_k}$ is an intercept term, and $M_l^{\vartheta_k}(\boldsymbol{\xi}_i)$ are smooth functions of covariates $\boldsymbol{\xi}_i$.

Basis Function Expansion Each smooth function $M_l(\boldsymbol{\xi}_i)$ can be represented as a linear combination of basis functions:

$$M_l(\boldsymbol{\xi}_i) = \sum_{m_l=1}^{M_l} \beta_{l,m_l} B_{l,m_l}(\boldsymbol{\xi}_i)$$

This allows the entire predictor vector $\boldsymbol{\gamma} = (\gamma_1, \ldots, \gamma_n)^T$ to be written as:

$$\boldsymbol{\gamma} = \beta_0 \mathbf{1}_n + \mathbf{Z}_1 \boldsymbol{\beta}_1 + \cdots + \mathbf{Z}_L \boldsymbol{\beta}_L$$

where $\mathbf{1}_n$ is a vector of ones, $\mathbf{Z}_l$ are design matrices, and $\boldsymbol{\beta}_l$ are coefficient vectors.

A2. Full Likelihood Derivation

Log-Likelihood Function For a random sample of n observations, the log-likelihood of our copula model is:

$$\ell(\boldsymbol{\beta}) = \sum_{i=1}^{n} \left[(1-y_{i1})\log\left\{F_{1|2}(0|y_{i2})\right\} + y_{i1}\log\left\{1-F_{1|2}(0|y_{i2})\right\} + \log f_2(y_{i2})\right]$$

where the conditional distribution function is:

$$F_{1|2}(0|y_{i2}) = \frac{\partial C(F_1(0), F_2(y_{i2});\theta)}{\partial F_2(y_{i2})} \tag{1}$$

Copula Definitions The specific copula functions used in this study are:

Gaussian Copula:

$$C(u,v;\theta) = \Phi_2(\Phi^{-1}(u), \Phi^{-1}(v);\theta)$$

Clayton Copula:

$$C(u,v;\theta) = \left(u^{-\theta} + v^{-\theta} - 1\right)^{-1/\theta}, \quad \theta > 0$$

Gumbel Copula:

$$C(u,v;\theta) = \exp\left[-\left((-\log u)^{\theta} + (-\log v)^{\theta}\right)^{1/\theta}\right], \quad \theta \geq 1$$

Frank Copula:

$$C(u,v;\theta) = -\frac{1}{\theta}\log\left[1 + \frac{(e^{-\theta u}-1)(e^{-\theta v}-1)}{e^{-\theta}-1}\right], \quad \theta \neq 0$$

A3. Score Functions and Analytical Derivatives

General Structure The score functions have a modular structure where:

- Two components depend on the chosen copula function
- Three terms depend on the marginal distributions
- One derivative depends on the link function

Detailed Score Equations **For the binary outcome parameter π:**

$$\frac{\partial \ell(\boldsymbol{\beta})}{\partial \boldsymbol{\beta}^{\pi}} = \sum_{i=1}^{n}\left[\frac{1-y_{i1}}{F_{1|2}(0|y_{i2})} - \frac{y_{i1}}{1-F_{1|2}(0|y_{i2})}\right] \cdot \frac{\partial F_{1|2}(0|y_{i2})}{\partial F_1(0)} \cdot \frac{\partial F_1(0)}{\partial \gamma_i^{\pi}} \cdot \mathbf{Z}^{\pi}[i,]$$

For the continuous outcome mean μ:

$$\frac{\partial \ell(\boldsymbol{\beta})}{\partial \boldsymbol{\beta}^{\mu}} = \sum_{i=1}^{n}\Bigg[\left(\frac{1-y_{i1}}{F_{1|2}(0|y_{i2})} - \frac{y_{i1}}{1-F_{1|2}(0|y_{i2})}\right)\frac{\partial F_{1|2}(0|y_{i2})}{\partial F_2(y_{i2})}\frac{\partial F_2(y_{i2})}{\partial \mu_i} + \frac{1}{f_2(y_{i2})}\frac{\partial f_2(y_{i2})}{\partial \mu_i}\Bigg]\frac{\partial \mu_i}{\partial \gamma_i^{\mu}}\mathbf{Z}^{\mu}[i,]$$

similar structures apply for the estimation of variance parameter σ^2 and copula parameter θ. Derivatives with respect to θ require evaluation of the copula density and its gradient. Closed-form expressions were used when available (Gaussian, Gumbel, Frank, Clayton). For cases where derivatives are algebraically complex, numerical differentiation was applied.

A4. Computational Implementation

Link Functions

- $\pi_i = \Phi(\gamma_i^\pi)$ (Probit link)
- $\mu_i = \gamma_i^\mu$ (Identity link)
- $\sigma_i = \exp(\gamma_i^\sigma)$ (Log link)

Numerical Optimization

- **Algorithm:** Trust-region Newton-Raphson
- **Convergence criterion:** Relative gradient $< 10^{-8}$
- **Hessian approximation:** Analytical where available, numerical otherwise
- **Software:** Implemented in R using custom C++ extensions for performance

A5. Model Selection

Information Criteria

$$\text{AIC} = -2\ell(\hat{\boldsymbol{\beta}}) + 2p, \quad \text{BIC} = -2\ell(\hat{\boldsymbol{\beta}}) + p\log(n)$$

where p is the total number of parameters and n is sample size.

References

Nadja Klein, Thomas Kneib, Giampiero Marra, Rosalba Radice, Slawa Rokicki, and Mark E McGovern. Mixed binary-continuous copula regression models with application to adverse birth outcomes. *Statistics in medicine*, 38(3):413–436, 2019.

Giampiero Marra and Rosalba Radice. Generalised joint regression modelling. *R Package*, 2017.
