# Supplementary material for: Unveiling the Multifaceted Dynamics of Breast Cancer: A Copula Regression Approach to Modeling and Predicting Outcomes
Source: PLoS One. 2026 Apr 10;21(4):e0346495. doi: 10.1371/journal.pone.0346495 (PMC13068339; doi:10.1371/journal.pone.0346495)
Supplement: Attachment — Submitted filename: Response_to_Reviewers_.pdf [file pone.0346495.s008.pdf]

Response to Reviewers

Manuscript ID: PONE-D-25-56986
Title: Unveiling the Multifaceted Dynamics of Breast Cancer: A Copula Regression Approach to Modeling and Predicting Outcomes

We sincerely thank the Academic Editor and the Reviewers for their careful evaluation of our manuscript and for their constructive and insightful comments. We have revised the manuscript extensively to improve clarity, methodological transparency, and interpretative precision.

All changes have been incorporated in the revised manuscript and are highlighted in the marked-up version. Detailed point-by-point responses are provided below.

Response to Reviewer #1

Comment 1: *"Define the survival endpoint precisely and justify binarizing it. If survival time and censoring are available, consider a time-to-event formulation or clearly motivate why it's not used."*

Response: We thank the reviewer for this important comment. In the revised manuscript, overall survival is now explicitly defined as a binary indicator coded as 0 if the patient was deceased (event) and 1 if the patient was alive at the last clinical follow-up (censored), irrespective of cause of death. This coding is consistent with the original METABRIC clinical annotation and was verified by cross-referencing with the variable `death_from_cancer` (see Methodology, page 4, lines 158–159).

Although time-to-event information is available in the METABRIC dataset, the primary objective of this study is to demonstrate copula-based joint modeling of mixed binary–continuous outcomes. Accordingly, a binary survival endpoint was adopted to facilitate joint modeling within a copula framework. This methodological focus and its scope are now explicitly clarified in the revised manuscript (see Methodology, pages 4–5, lines 160–167).

Comment 2: *"Justify treating age at diagnosis as a response; otherwise, move age to the predictor side and focus the joint model on outcomes that are truly co-modeled."*

Response: We agree that age at diagnosis temporally precedes survival. In the revised manuscript, we explicitly clarify that age is not treated as a response in a predictive or causal sense. Rather, it is modeled as a continuous marginal outcome solely to enable joint modeling with survival status and to quantify their dependence structure within a copula framework.

During exploratory analyses, several clinically relevant outcome pairings were examined (e.g., survival jointly modeled with tumor size and with the Nottingham Prognostic Index). These combinations exhibited negligible dependence. In contrast, the age–survival pairing demonstrated non-negligible dependence across multiple copula families, motivating its selection for detailed analysis.

The manuscript now explicitly states that no causal or temporal interpretation is implied (see Study Variables subsection, page 4, lines 170–175).

Comment 3: *"Remove or heavily qualify interpretations that look like survival 'explains' age. Present Equation (2) as a symmetric association component (not causal) or restructure the model."*

Response: We appreciate the reviewer's careful reading. All language suggesting causal or directional relationships has been removed. Interpretations associated with Equation (2) now explicitly emphasize that the copula component represents a symmetric association between outcomes

without implying causality.

These clarifications have been incorporated in the methodological description and in the interpretation of results (see Subsection: Modeling Mixed Binary and Continuous Outcomes, page 5, lines 185–194). **Comment 4:** *"Correct the Kendall's τ/θ inconsistency for the Clayton model and ensure the narrative matches Tables 2–3."*

Response: We thank the reviewer for this careful observation. The inconsistency between Kendall's τ and θ for the Clayton copula has been corrected. The revised values now satisfy the theoretical relationship $\tau = \theta/(\theta + 2)$. For the Clayton copula with $\theta = 28$, this gives $\tau = 28/30 = 0.933$, which now matches Table 3. The narrative has been updated accordingly to ensure consistency throughout the manuscript (see Tables 2–3, pages 14–15).

Comment 5: *"Clarify the full model specification, which covariates enter π, μ, σ^2, and θ. If θ is constant, remove claims about covariate dependent dependence (or report those results)."*

Response: We thank the reviewer for this important clarification. The full model specification has now been made explicit. The probability of survival (π) includes covariates such as age, ER status, PR status, HER2 status, chemotherapy, hormone therapy, radiotherapy, Nottingham Prognostic Index, Positive lymph node , and tumor size. The mean age (μ) includes overall survival, histologic grade, Nottingham Prognostic Index, tumor size, lymph node status, ER status, and PR status. The variance (σ^2) is modeled with only an intercept. The copula dependence parameter (θ) is specified as constant within each copula family, capturing the overall dependence between outcomes without covariate dependence.

All claims suggesting covariate-dependent θ have been removed, and the constant specification is now clearly stated throughout the manuscript (see Regression Framework and Estimation subsection, page 22, lines 322–353 and Appendix).

Response to Reviewer #2

Comment 1: *"How exactly is overall survival defined in the study and why reduce it to a binary outcome if time-to-event data exist?"*

Response: Overall survival is defined as a binary indicator equal to 1 if the patient was alive at last follow-up (censored) and 0 if deceased (event), regardless of cause of death, as recorded in the METABRIC dataset. This definition is now explicitly stated in the revised manuscript (page 4, lines 158–159).

The binary formulation was adopted to support the study's primary objective of joint modeling mixed binary–continuous outcomes using copula regression. While time-to-event modeling is important, it lies beyond the methodological scope of the present study. This scope is now clearly acknowledged.

Comments 2 and 3: *"Why is age at diagnosis treated as a response variable?"*
"How should readers interpret 'Overall Survival (Yes)' predicting age without implying reverse causality?"

Response: Age at diagnosis is included as a continuous marginal outcome solely to facilitate joint modeling with survival status and to quantify their dependence structure. The model does not imply that survival predicts age, nor does it impose any reverse causality. The copula framework captures symmetric association between outcomes without imposing causal or temporal direction.

Relevant language has been revised throughout the manuscript to ensure interpretive clarity (see Study Variables subsection, page 4, lines 170–175; and Model Specification, page 5, lines 185–194).

Comment 4: *"This study shows that Clayton Kendall's $\tau = 0.60$ for $\theta = 28$, but Table 3 reports $\tau = 0.933$."*

Response: We appreciate the reviewer's careful observation. The inconsistency between Kendall's τ and θ for the Clayton copula has been corrected. The revised values now satisfy the theoretical relationship $\tau = \theta/(\theta + 2)$. Tables 2–3 and the corresponding text have been updated accordingly.

Comment 5: *"This study states that the copula parameter θ is linked to covariates. Was θ actually modeled as covariate dependent, or is it constant (as the single θ per model suggests)?"*

Response: We thank the reviewer for pointing this out. We clarify that although the general copula regression framework allows the dependence parameter θ to be linked to covariates, in the present study, θ was modeled as a constant parameter within each copula family. This approach captures the overall strength of dependence between the two outcomes, while covariates influence the marginal distributions of survival (π) and age (μ, σ^2).

We have revised the manuscript in Section "Regression Framework and Estimation" (Page 6, Lines 220–227) to explicitly state that all results reported correspond to models with a constant θ. Covariate-dependent dependence structures were explored only in preliminary analyses and are beyond the scope of the present paper.

Comment 6: *"Tumor size shows a positive association with 'survival' in Table 2. Does this reflect coding, collinearity (e.g., with NPI), or scaling issues?"*

Response: We thank the reviewer for this important observation. Given our coding (1 = alive, 0 = deceased), the positive coefficient indicates that larger tumor size was associated with a higher modeled probability of being alive after covariate adjustment. This counterintuitive result was not consistently significant across copula families (see Tables 2–3), suggesting it is a model-specific conditional association rather than a robust marginal effect. We have revised the interpretation

to clarify its conditional and model-specific nature (Subsection “Interpretation of Copula Model Results,” Page 12, Lines 355–363).

Comment 7: *“Beyond AIC/BIC, did the authors run any copula diagnostics or compare against an independent margins baseline?”*

Response: We thank the reviewer for this valuable suggestion. We have added comprehensive diagnostic analyses:

1. **Independent margins baseline:** Separate probit and Gaussian linear models were fitted and compared with the joint Gumbel copula model. A likelihood ratio test strongly rejects independence ($\chi^2 = 2190.24$, df = 1, $p < 0.0001$). Both AIC and BIC strongly favor the copula specification (ΔAIC = 2,168, ΔBIC = 2,084), demonstrating substantial dependence between outcomes.

2. **PIT diagnostics:** Marginal adequacy was assessed using Probability Integral Transform (PIT) residuals. For the continuous age margin, the PIT histogram showed acceptable uniformity (Kolmogorov–Smirnov test: $D = 0.027$, $p = 0.126$). For the binary survival margin, a randomized PIT approach was employed to accommodate discreteness (Czado et al., 2009); resulting residuals were approximately uniform (KS test: $D = 0.022$, $p = 0.301$).

These diagnostics are now included in the Results section under “Model Diagnostics and Comparison” (pages 13–14, lines 404–430).

Comment 8: *“Tumor size is reported in mm in Table 1, but appears as cm in the regression tables.”*

Response: Thank you for pointing out this inconsistency. We have carefully checked the units and corrected the regression tables so that tumor size is consistently reported in millimeters (mm) throughout the manuscript. All tables now use uniform units.

Comment 9: *“All figures should be replotted with better resolution.”*

Response: We agree and have replotted all figures using higher-resolution graphics suitable for publication (300 dpi, TIFF format with LZW compression). The updated figures have been uploaded separately in the submission system to ensure clarity in print and online versions.

Comment 10: *“The literature review lacks many applications of copula regression model for analysis and prediction in different fields. See: Modeling car-following behaviors using a driving style–based Bayesian model averaging Copula framework in mixed traffic flow. Jointly analyzing freeway traffic incident clearance and response time using a copula-based approach.”*

Response: We appreciate this constructive suggestion. The Introduction has been substantially expanded to include diverse applications of copula methods across disciplines. Specifically, we now discuss applications in:

- Transportation research (including the studies suggested by the reviewer: incident clearance analysis by Zou et al., 2018, and car-following behaviors)
- Finance and economics (Patton, 2006; Clementi and Gianmoena, 2017)
- Environmental science and hydrology (Salvadori and De Michele, 2007; Zhang et al., 2013, 2017)
- Agricultural economics (Larsen et al., 2013)

- Biomedical and cancer research applications (Gasparini and Humphreys, 2022; Emura and Chen, 2018)

More than 15 additional references have been incorporated to strengthen the contextual foundation of the study while maintaining focus on our methodological contribution to cancer research.

We thank both reviewers for their time and expertise, which have substantially improved our manuscript. We believe the revisions address all concerns raised and hope that the manuscript is now suitable for publication in PLOS ONE.
